# Development and thyroid hormone dependence of skeletal muscle mitochondrial function towards birth

**DOI:** 10.1113/JP279194

**Published:** 2020-03-27

**Authors:** K. L. Davies, E. J. Camm, E. V. Atkinson, T. Lopez, A. J. Forhead, A. J. Murray, A. L. Fowden

**Affiliations:** ^1^ Department of Physiology Development and Neuroscience University of Cambridge Cambridge CB2 3EG UK; ^2^ Department of Biological and Medical Sciences Oxford Brookes University Oxford OX3 0BP UK

**Keywords:** birth, development, hypothyroidism, mitochondria

## Abstract

**Key points:**

- Skeletal muscle energy requirements increase at birth but little is known regarding the development of mitochondria that provide most of the cellular energy as ATP.
- Thyroid hormones are known regulators of adult metabolism and are important in driving several aspects of fetal development, including muscle fibre differentiation.
- Mitochondrial density and the abundance of mitochondrial membrane proteins in skeletal muscle increased during late gestation. However, mitochondrial functional capacity, measured as oxygen consumption rate, increased primarily after birth.
- Fetal hypothyroidism resulted in significant reductions in mitochondrial function and density in skeletal muscle before birth.
- Mitochondrial function matures towards birth and is dependent on the presence of thyroid hormones, with potential implications for the health of pre‐term and hypothyroid infants.

**Abstract:**

Birth is a significant metabolic challenge with exposure to a pro‐oxidant environment and the increased energy demands for neonatal survival. This study investigated the development of mitochondrial density and activity in ovine biceps femoris skeletal muscle during the perinatal period and examined the role of thyroid hormones in these processes. Muscle capacity for oxidative phosphorylation increased primarily after birth but was accompanied by prepartum increases in mitochondrial density and the abundance of electron transfer system (ETS) complexes I–IV and ATP‐synthase as well as by neonatal upregulation of uncoupling proteins. This temporal disparity between prepartum maturation and neonatal upregulation of mitochondrial oxidative capacity may protect against oxidative stress associated with birth while ensuring energy availability to the neonate. Fetal thyroid hormone deficiency reduced oxidative phosphorylation and prevented the prepartum upregulation of mitochondrial density and ETS proteins in fetal skeletal muscle. Overall, the data show that mitochondrial function matures over the perinatal period and is dependent on thyroid hormones, with potential consequences for neonatal viability and adult metabolic health.

## Key points

- Skeletal muscle energy requirements increase at birth but little is known regarding the development of mitochondria that provide most of the cellular energy as ATP.
- Thyroid hormones are known regulators of adult metabolism and are important in driving several aspects of fetal development, including muscle fibre differentiation.
- Mitochondrial density and the abundance of mitochondrial membrane proteins in skeletal muscle increased during late gestation. However, mitochondrial functional capacity, measured as oxygen consumption rate, increased primarily after birth.
- Fetal hypothyroidism resulted in significant reductions in mitochondrial function and density in skeletal muscle before birth.
- Mitochondrial function matures towards birth and is dependent on the presence of thyroid hormones, with potential implications for the health of pre‐term and hypothyroid infants.

## Introduction

Birth is a significant metabolic challenge to the neonate. It must maintain its internal environment for the first time and activate many vital processes that have little or no function *in utero*, including pulmonary respiration, enteral nutrition, gluconeogenesis and thermoregulation (Fowden *et al*. 1998). Consequently, neonatal energy demands rise rapidly after birth as organs and tissues assume these new roles. The energy needed is provided as ATP, produced primarily by oxidative phosphorylation (OXPHOS) in the mitochondria, which requires and accounts for the significant increase in the rate of oxygen consumption after birth (Klein *et al*. 1983). This, together with the fluctuations in arterial partial pressure of oxygen ($P_{aO_{2}}$ ) during labour and the rapid rise in O_2_ exposure at birth, increases the risk of excessive mitochondrial superoxide production and oxidative damage to the neonatal tissues (Rogers *et al*. 1998). Preparations for the neonatal metabolic challenges begin before birth in several of the key tissues essential for neonatal survival, but little is known regarding the normal prepartum maturation of mitochondrial function in any fetal tissue.

In adults, mitochondria are highly dynamic organelles and respond to changes in cellular energy demands by biogenesis/mitophagy, by fusion/fission, and by alterations in the abundance of the electron transfer system (ETS) and other proteins regulating ATP and superoxide production (Nunnari & Suomalainen, 2012; Schrepfer & Scorrano, 2016). With substrates provided via the tricarboxylic acid cycle and other intermediary pathways, ATP is synthesised from ADP by ATP synthase using the proton gradient across the inner mitochondrial membrane (IMM) generated by the redox reactions at the ETS protein complexes (Nunnari & Suomalainen, 2012; Schrepfer & Scorrano, 2016). The electrons derived from the reducing equivalents pass along the ETS to produce water using O_2_ but they can also be diverted from the ETS to form superoxide, particularly when the proton gradient is high or there are abnormalities in the O_2_ or substrate supply (Aledo, 2014). In addition, mitochondrial respiratory function is regulated, in part, by uncoupling proteins (UCPs), which dissipate the proton gradient, thereby decreasing superoxide production but also OXPHOS efficiency (Adjeitey *et al*. 2013). Furthermore, ATP production depends on ADP availability which is controlled by the IMM transporter adenine nucleotide translocase (ANT) using the proton gradient (Brand *et al*. 2005). Relatively little is known about the ontogenic changes in any of these mitochondrial proteins during the perinatal period, although alterations in UCP expression have been observed during late gestation in the fetal lung and adipose tissue (Mostyn *et al*. 2003; Gnanalingham *et al*. 2005).

With the onset of pulmonary respiration, locomotion and shivering thermogenesis at birth, skeletal muscle makes a significant contribution to the increased energy demand and total rate of O_2_ consumption seen neonatally, particularly in precocial species such as sheep which are mobile shortly after birth. Skeletal muscles generally consist of a mixture of fibre types which are subdivided into two main types: slow twitch, oxidative type I fibres with abundant mitochondria, and fast twitch type II fibres with fewer mitochondria and which carry out oxidative/glycolytic (type IIa) or predominantly glycolytic metabolism (type IIx; Yates *et al*. 2016). These fibre types can be identified by their expression of the different myosin heavy chain (MHC) isoforms, MHCI, MHCIIa and MHCIIx (Yates *et al*. 2016). The majority of skeletal muscle fibres are formed during mid‐gestation with both slow and fast twitch fibres present by late gestation in precocial species (Javen *et al*. 1996). However, the specific fibre composition of individual muscles reflects their function, neuronal stimulation and the environmental conditions during intrauterine development (Gambke *et al*. 1983).

Many of the prepartum maturational processes essential for neonatal survival depend on hormonal changes in the fetus in the period immediately before birth. In particular, the increases in fetal cortisol and tri‐iodothyronine (T_3_) concentrations towards term are important for the successful transition to extrauterine life in several species, including sheep and humans (Fowden *et al*. 1998; Forhead & Fowden, 2014). Thyroid hormones also affect metabolic rate and mitochondrial function through actions on mitochondrial biogenesis as well as UCP and ANT expression in adult tissue, including skeletal muscle (Dummler *et al*. 1996; Barbe *et al*. 2001; Wulf *et al*. 2008; Lombardi *et al*. 2015). In addition, in species that are immature at birth (e.g.rodents), thyroid hormones are known to influence the maturational development of muscle morphology and bioenergetics in the period after birth (Lombardi *et al*. 2015; Bloise *et al*. 2018). However, much less is known about the metabolic role of thyroid hormones *in utero*, although the whole body rate of O_2_ consumption is positively correlated to thyroxine (T_4_) concentrations in fetal sheep during late gestation (Fowden & Silver, 1995). Previous studies have also shown that thyroid hormones can affect the fibre phenotype of fetal ovine muscles and the rate of O_2_ consumption by specific porcine muscles around the time of birth (Finkelstein *et al*. 1991; Herpin *et al*. 1996). However, little is known about the role of thyroid hormones in the development of skeletal muscle mitochondria during the perinatal period when T_3_ concentrations are rising most rapidly in preparation for extrauterine life. The current study therefore examined the ontogeny of mitochondrial function in ovine skeletal muscle during late gestation and early neonatal life and tested the hypothesis that any ontogenic changes would be dependent on fetal thyroid hormone availability.

## Materials and methods

All animal procedures were regulated by project and personal licences under the UK Animals (Scientific Procedures) Act 1986 Amendment Regulations 2012 after ethical review by the University of Cambridge Animal Welfare and Ethical Review Body (UBS: AWERB.0902.2018.1A). All investigators understood and worked by the ethical principles and standards discussed by Grundy (2015).

### Experimental procedures

Eighteen Welsh Mountain ewes bearing twins and six newborn twin lambs were used in this study. Ewes bought from commercial sheep sales were transported to University of Cambridge designated premises according to the UK's Live transport: welfare regulations 2012. They were mated and maintained on site both before and during the experimental procedures. All pregnant and lactating ewes were group housed and had free access to hay and water. At 102–105 days of gestational age after fasting for 18–24 h (dGA; term ∼145 dGA), 12 of the pregnant ewes underwent surgery in which one twin fetus was thyroidectomised (TX) while the other was sham‐operated under isofluorane anaesthesia (1.5–2% in 5:1 O_2_/N_2_O mixture) as described previously (Forhead *et al*. 2002). A pulse oximeter and capnograph were used to monitor maternal heart rate, $P_{O_{2}}$ and $P_{CO_{2}}$ throughout surgery with the oxygen flow rate and percentage isofluorane adjusted accordingly. Ewes received analgesia (1 mg kg^−1^ carprofen, s.c.) and antibiotics (oxytetracycline, 20 mg kg^−1^ i.m. and penicillin, 15 mg kg^−1^ i.m. to mother and i.a. to fetus) on the day of surgery with maternal penicillin treatment continuing for two further days. These ewes and their fetuses were killed for tissue collection at either 126–129 dGA (*n* = 6 ewes) or 140–145 dGA(*n* = 6 ewes). The order of delivery of the TX or sham‐operated fetus was randomised, as the uterine horn containing each fetus was known from surgery. Tissue was also collected after euthanasia from the fetuses of the remaining six unoperated twin pregnant ewes at 102–105 dGA and from six unoperated newborn twin lambs at 1–2 days of postnatal age. Only one of each pair of unoperated twins was selected randomly for further study to ensure a mixture of sexes and order of delivery, where relevant. The male to female ratios in the groups were as follows: 104 dGA 2:4; 127 dGA sham 3:3; 127 dGA TX 3:3; 143 dGA sham 2:4; 143 dGA TX 2:4; newborns 3:3. All ewes, fetuses and newborn lambs were killed via an overdose of anaesthetic (200 mg kg^−1^ sodium pentobarbitone, i.v.). Blood samples were taken from the umbilical artery of the fetuses or from jugular vein of the lambs and centrifuged in heparin‐coated tubes before plasma storage at −20°C for future hormone analysis.

After euthanasia, the fetus or lamb was weighed and biometric measurements were taken including crown–rump length (CRL) and limb lengths. The biceps femoris (BF) was immediately collected from the left leg and weighed. A portion from the centre of the muscle (Kohn & Myburgh, 2007) was collected into ice cold biopsy preservation medium (BIOPS; pH 7.1 solution containing 10 mm Ca‐EGTA buffer, 0.1 μm free Ca^2+^, 1 mm free Mg^2+^, 20 mm imidazole, 20 mm taurine, 50 mm K‐MES, 0.5 mm dithiothreitol, 6.56 mm MgCl_2_, 5.77 mm ATP and 15 mm phosphocreatine; Pesta & Gnaiger, 2012) for respirometry. A second portion of the muscle was fix‐frozen for immunohistochemistry by immersion in PBS before transferring to pre‐cooled isopentane. The remaining tissue was snap‐frozen in liquid nitrogen and the fix‐frozen tissue was stored at −80°C until required.

### Biochemical analyses

#### Hormone assays

Cortisol concentrations were measured using a commercial human enzyme‐linked immunoassay (IBL International, Hamburg, Germany), validated for use with sheep plasma (Vaughan *et al*. 2016). Inter‐assay variation was 4.80% and intra‐assay variation was 2.65%. The limit of detection of the assay was 5.2 ng ml^−1^. Total plasma T_3_ and T_4_ concentrations were measured using radioimmunoassays (MP Biomedical, Santa Ana, CA, USA) validated for use with sheep plasma (Fowden & Silver, 1995). For T_3_, inter‐assay variation was 7.59% and intra‐assay variation was 2.28%. The limit of detection of the assay was 0.14 ng ml^−1^. For T_4_, inter‐assay variation was 4.57% and intra‐assay variation was 3.04%. The limit of detection of the assay was 11.3 ng ml^−1^.

#### Skeletal muscle composition

Sections of muscle were weighed, freeze‐dried for 24 h and re‐weighed in order to calculate percentage water content. Total protein was extracted from frozen fetal skeletal muscle samples (55 mg ± 10%), and concentration was measured using a bicinchoninic acid assay. Protein content was expressed as mg protein per gram tissue (wet weight) or as mg protein per mg dry weight depending on the measurement to be normalised.

#### Respirometry

Respirometry was carried out as described previously for skeletal muscle samples (Kuznetsov *et al*. 2008; Pesta & Gnaiger, 2012). Briefly, muscle samples in ice cold BIOPS were dissected into 2–3 mg pieces, fibres were teased apart and plasma membranes were permeabilised (100 μg saponin ml^–1^ BIOPS; 20 min). Oxygen consumption of muscle fibre bundles was measured in an isotonic sucrose‐based respiration medium (MiR05; pH 7.1 solution containing 20 mm HEPES, 0.46 mm EGTA, 2.1 mm free Mg^2+^, 90 mm K^+^, 10 mm P_i_, 20 mm taurine, 110 mm sucrose, 60 mm lactobionate and 1 g l^−1^ BSA; Pesta & Gnaiger, 2012) which was developed to optimise mitochondrial respiration in *ex vivo* preparations at 37°C (Gnaiger *et al*. 2000). Oxygen consumption was measured using Clark‐type oxygen electrodes (Strathkelvin Instruments, Glasgow, UK). Mitochondrial respiratory studies were all carried out at 37°C in line with standard practice for mammalian respirometry measurements. The use of a single temperature allowed us to make direct comparisons of mitochondrial respiratory capacity across all groups, which would not otherwise have been possible owing to effects of temperature on oxygen electrode behaviour and oxygen saturation in the respiratory medium.

After a baseline period, substrates were introduced into the chambers according to three different protocols. First, 2 mm malate plus 10 mm glutamate were added to saturate electron entry via complex I of the ETS, initially without ADP (leak state) and then with 10 mm ADP (OXPHOS state), before 10 mm succinate was added to additionally support electron entry via complex II and thereby saturate electron entry to the electron transport chain. In the two subsequent assays, more physiological substrates were provided to probe substrate‐led pathways. Accordingly, in the second assay, 2 mm malate plus 5 mm pyruvate ± ADP, was provided to probe the capacity for the oxidation of pyruvate, which is derived from glucose *in vivo* via glycolysis and metabolised in the mitochondria by pyruvate dehydrogenase, supporting electron transport through the generation of NADH. Finally, in the third assay, 2 mm malate plus 40 μm palmitoyl carnitine (PC) ± 10 mm ADP were added to saturate β‐oxidation and indicate the capacity for mitochondrial fatty acid oxidation. All three protocols concluded with the addition of 10 μm cytochrome c to check the integrity of the outer mitochondrial membrane. Results were excluded if there was an increase in O_2_ consumption of 15% or more upon cytochrome c addition, or if the rate of O_2_ uptake over the baseline period, before substrates were added, exceeded 0.001 μmol O_2_ min^–1^ indicating insufficient plasma membrane permeabilisation (Kuznetsov *et al*. 2008). The samples were dried at 80°C for 48 h and weighed to normalise the respiration rates to tissue dry weight. Results are given as O_2_ consumption mg^–1^ dry weight.

#### Enzyme assays

Citrate synthase (CS) activity of 30 μg homogenised BF protein was measured spectrophotometrically at 37°C. The assay buffer (pH 8) contained 0.1 mm 5,5′‐dithio‐bis(2‐nitrobenzoic acid) (DTNB), 1 mm oxaloacetate and 0.3 mm acetyl‐CoA. The maximal rate of change of absorbance at 412 nm [rate of 5‐thio‐2‐nitrobenzoic acid (TNB) production] over a 3 min period was used to determine CS activity, expressed per mg protein. To directly normalise respirometry data to CS activity, CS activity was initially converted to activity mg^–1^ dry weight using water and protein content values.

β‐Hydroxyacyl‐CoA dehydrogenase (HOAD) activity of 20 μg homogenised muscle protein was measured spectrophotometrically at 37°C. The assay buffer (pH 7.4) contained 0.15 mm NADH and 0.1 mm acetoacetyl‐CoA. The rate of change of 340 nm absorbance (oxidation of NADH to NAD^+^) over the following 3 min period was used to determine HOAD activity.

#### Western blotting

Total protein was extracted from frozen BF samples (55 mg ± 10%), diluted to 2.5 μg μl^−1^ in 8% SDS solution and electrophoresed on a 12% polyacrylamide gel. Protein was transferred to nitrocellulose membrane and stained using Ponceau‐S for normalisation of protein loading. Membranes were probed with primary antibodies to ETS complexes (OXPHOS antibody cocktail; Life Technologies, Carlsbad, CA, USA; 458099; 1:1000; RRID: AB_2533835), followed by HRP‐linked secondary antibody (GE Healthcare, Amersham, UK; NIF82; 1:5000). Protein bands were visualised using enhanced chemiluminescence for quantification using ImageJ (http://rsb.info.nih.gov/ij/).

#### Gene expression analyses

RNA was extracted from frozen skeletal muscle using TRIzol (Thermo Fisher Scientific, Invitrogen, Waltham, MA, USA) and chloroform and the aqueous phase was separated and used in an RNeasy Plus kit (Qiagen, Manchester, UK). The concentration of the eluted RNA was measured using a Nanodrop ND‐1000 spectrophotometer, diluted to 50 ng μl^−1^ and reverse transcribed to cDNA (High Capacity cDNA Reverse Transcription Kit; Applied Biosystems, Foster City, CA, USA). Quantitative real‐time PCR (qRT‐PCR) was performed using a MESA BLUE Mastermix (Eurogentec, Seraing, Belgium) on a 7500 Fast Real‐Time PCR System (Applied Biosystems) following the recommended protocol for SYBR: an initial denaturation step (5 min at 95°C) was followed by 40 amplification cycles (15 s at 95°C and 1 min at 60°C). The primer sequences are given in Table 1. Data were analysed using the 2^−ΔΔCt^ method (Schmittgen & Livak, 2008) and expressed relative to the mRNA levels of the housekeeper ribosomoal protein *S15*, and set relative to 1 experimental sample. The product size from each primer pair was checked by running on an agarose gel.

**Table 1:** Forward and reverse primer sequences used for SYBR qRT‐PCR

Target gene and protein	Primer sequences	Reference
*RPS15*	F: ATCATTCTGCCCGAGATGGTG	Yates *et al*. (2016)
*Ribosomal protein S15*	R: TGCTTCACGGGCTTGTAGGTG	
*Myosin heavy chain 7* (*MHY7*)	F: GAGATGGCCGCGTTTGGGGAG	Yates *et al*. (2016)
MHCI protein	R: GGCTCGTGCAGGAAGGTCAGC	
*MHY2*	F: ACCGAAGGAGGGGCGACTCTG	Yates *et al*. (2016)
MHCIIa protein	R: GGCTCGTGCAGGTGGGTCATC	
*MYH1*	F: AAAGCGACCGTGCAGAGCAGG	Yates *et al*. (2016)
MHCIIx protein	R: GGCTCGTGCAGGTGGGTCATC	
*Peroxisome proliferator‐activated receptor gamma coactivator 1 alpha* (*PPARGC1A*)	F: GAGATGTGACCACCGAGAATGAG	Myers *et al*. (2008)
PGC1α protein	R: GCTGTTGACAAATGCTCTTCGC	
*Mitofusin 2 (MFN2)*	F: CATCAGCTATACTGGCTCCAACT	This paper
MFN2 protein	R: AATGAGCAAAAGTCCCAGACA	
*Uncoupling protein 2 (UCP2)*	F: AAGGCCCACCTAATGACAGA	This paper
UCP2 protein	R: CCCAGGGCAGAGTTCATGT	
*UCP3*	F: GAAAGGAATTCTGCCCAACA	Kelly *et al*. (2011)
UCP3 protein	R: TCCAAAGGCAGAGACGAAGT	
*SLC25A4*	F: TGGTGTCCTACCCCTTTGAC	Kelly *et al*. (2011)
Adenine nucleotide translocase 1 (ANT1) protein	R: CAGGCGCCTTTGAAGAAAGC	

#### Immunohistochemistry

Transverse sections (10 μm) were cut from the fixed frozen samples and transferred to glass slides for immunostaining (Yates *et al*. 2016). Sections were fixed in acetone at −20°C, incubated in Tris‐buffered saline with Triton and Tris‐buffered saline containing 0.1% Tween‐20 and 0.1% Triton (TBS‐TT) followed by 3% H_2_O_2_ and blocked in TBS containing 5% goat serum and 2% bovine serum albumin. Primary antibody, diluted 1:20 in TBS‐TT, was applied overnight at 4°C: MHCI [BA‐D5; Developmental Studies Hybridoma Bank (DSHB), University of Iowa, Iowa City, IA, USA; RRID: AB_2235587] and MHCII (F18; DSHB; RRID: AB_528369) and fibres were counterstained with Desmin (Y66; Abcam, Cambridge, UK; 1:300; RRID: AB_731901). Alexa Fluor‐conjugated secondary antibodies, diluted 1:200, were used to visualise immunocomplexes (Alexa Fluor 488; Abcam ab150105; RRID: AB_2732856 and Alexa Fluor 568; Abcam ab175471; RRID: AB_2576207). An Axio Imager A1 microscope was used to take five images per section at 40× magnification and ImageJ was used to quantify the number and average fibre area of type I and II fibres in the field of view.

### Statistical analysis

All results are presented as mean ± SD and analyses were performed using GraphPad Prism Version 6.05 for Windows (www.graphpad.com). One‐way ANOVA was applied to the ontogeny data, followed by a Tukey's multiple comparison *post hoc* test as appropriate. A *t* test or Mann–Whitney non‐parametric test was used to compare control and TX values at the same age, and to compare the TX value at 143 dGA to that at 127 dGA. The Pearson product‐moment correlation coefficient was used to assess linear correlation between variables and log‐transformed hormone data. Partial correlation analyses were used to determine the relationship between two variables while controlling for a third variable. *P* < 0.05 was considered significant throughout.

## Results

### Hormones and biometry

Fetal plasma concentrations of cortisol and T_3_ showed a normal prepartum rise towards term (Table 2), consistent with previous studies (Forhead & Fowden, 2002; Mostyn *et al*. 2003). Both concentrations rose still further in the newborn lambs (one‐way ANOVA all four groups; Table 2). Plasma T_4_ concentration did not differ significantly with age (Table 2). There were no significant thyroid gland remnants in TX fetuses at post‐mortem and their plasma concentrations of T_3_ and T_4_ were significantly lower than control values at both gestational ages (Table 2). TX did not significantly alter fetal plasma cortisol concentrations at either age (Table 2).

**Table 2:** Fetal and neonatal hormonal and morphometric measurements and biceps femoris (BF) muscle structural data

	104dGA	127dGA		143dGA		Newborn
	Control	Control	TX	Control	TX	Control
Hormone concentrations						
Plasma cortisol (ng ml^−1^)	13.8 ± 2.9 ^aA^	12.1 ± 2.9 ^aA^	14.0 ± 6.5	38.2 ± 15.9 ^aB^	25.0 ± 14.4	122.0 ± 59.0 ^b^
Plasma T_3_ (ng ml^−1^)	0.33 ± 0.04 ^aA^	0.33 ± 0.03 ^aA,B^	0.21 ± 0.07^†^	0.60 ± 0.31 ^aB^	0.24 ± 0.04^*^	4.02 ± 0.81 ^b^
Plasma T_4_ (ng ml^−1^)	78.0 ± 23.1	104.9 ± 28.6	10.1 ± 5.1^‡^	87.8 ± 13.8	13.4 ± 16.1^†^	83.1 ± 19.0
Morphometry						
Body weight (kg)	1.19 ± 0.43 a	2.54 ± 0.25 ^b^	2.33 ± 0.33	3.49 ± 0.63 ^c^	3.25 ± 0.47	3.34 ± 0.34 ^c^
Crown–rump length (cm)	31.5 ± 2.2 ^a^	41.8 ± 1.9 ^b^	40.0 ± 1.3	45.0 ± 1.3 ^c^	44.0 ± 3.8	49.6 ± 2.4 ^d^
Femur (cm)	5.8 ± 0.9 ^a^	9.3 ± 1.1 ^b^	8.8 ± 0.4	10.3 ± 0.8 ^b^	9.8 ± 1.5	12.8 ± 1.1 ^c^
Tibia (cm)	8.8 ± 0.6 ^a^	12.3 ± 0.8 ^a,b^	11.7 ± 0.5	14.5 ± 1.3 ^b^	13.3 ± 1.3	12.4 ± 5.5 ^b^
Metatarsus (cm)	10.0 ± 0.6 ^a^	14.3 ± 0.6 ^a,b^	13.0 ± 0.6^†^	16.3 ± 0.8 ^b,c^	14.7 ± 1.0^*^	17.5 ± 0.8 ^c^
BF weight (g)	5.46 ± 0.97 ^a^	11.02 ± 1.12 ^b^	10.92 ± 2.18	13.91 ± 3.30 ^b,c^	13.97 ± 1.90	15.39 ± 2.95 ^c^
BF/body weight ratio (g kg^–1^)	4.86 ± 1.10	4.34 ± 0.19	4.71 ± 0.86	3.96 ± 0.38	4.30 ± 0.20^*^	4.61 ± 0.77
Biochemical composition						
BF water content (%)	86.8 ± 0.9 ^a^	83.3 ± 1.2 ^b^	85.8 ± 0.9^†^	79.3 ± 1.4 ^c^	83.5 ± 2.1^†^	78.6 ± 0.7 ^c^
BF protein content (mg g^–1^ wet wt)	44.5 ± 2.9 ^a^	45.7 ± 1.6 ^a^	40.3 ± 5.1^*^	48.3 ± 5.3 ^a^	45.1 ± 4.7	59.6 ± 4.7 ^b^

Data are presented as mean ± SD of fetuses at 104, 127 and 143 days of gestational age (dGA) and newborn lambs. *N* = 5–6 per measurement in each group. Values with different letters are significantly different from each other (*P* < 0.05 by Tukey's *post hoc* test following one‐way ANOVA); lower case letters include all four age groups, while upper case letters include 104–143 dGA groups only.

Significantly different from control at the same gestational age: ^*^ *P* < 0.05 (by *t* test); ^†^ *P* < 0.01; ^‡^ *P* < 0.0001.

Body weight, CRL, and the lengths of the femur, tibia and metatarsus increased significantly with age across the four groups, but only metatarsus length was affected by TX, being shorter than in the controls at both 127 and 143 dGA (Table 2). BF weight but not BF/body weight ratio increased significantly with age (Table 2). Muscle water content decreased with age while the protein content increased with age (Table 2). There was no effect of TX on the BF weight although BF/body weight was greater in TX than control fetuses at 143 dGA (Table 2). Muscle of TX fetuses had higher water content than controls, and protein content was lower in BF of TX than control fetuses at 127 dGA (Table 2).

### Muscle oxygen consumption

**Figure 1:**
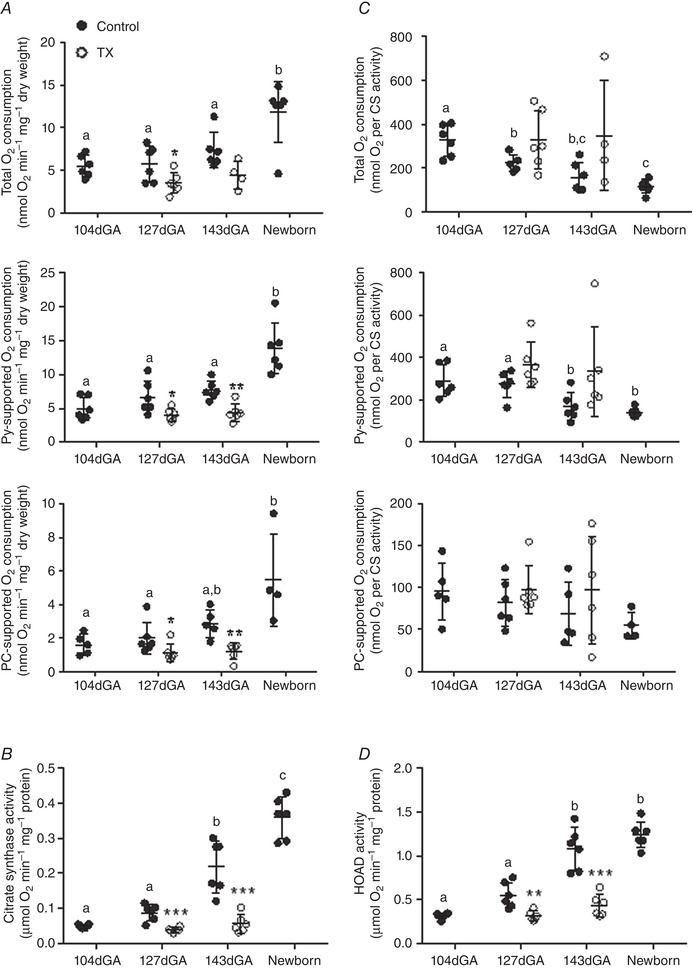
Functional oxidative capacity of late gestation ovine biceps femoris muscle Values are mean ± SD. *A*, ADP‐coupled O_2_ consumption during maximal respiration (malate, glutamate and succinate‐driven; total), respiration supported by pyruvate (Py) plus malate and palmitoyl carnitine (PC) plus malate. *B*, citrate synthase (CS) activity and *C*, O_2_ consumption normalised to CS activity as labelled. *D*, β‐hydroxyacyl‐CoA dehydrogenase (HOAD) activity. Data are presented for fetuses at 104, 127 (control and TX) and 143 days of gestational age (dGA; control and TX) and newborn lambs (*n* = 4–6 per group). Control data are represented by filled circles and thyroidectomy (TX) data by open circles. Different letters indicate significant difference to each other (*P* < 0.05 by Tukey's *post hoc* test following one‐way ANOVA). Asterisks indicate significantly different from control at the same gestational age: ^*^ *P* < 0.05 (by *t* test); ^**^ *P* < 0.01; ^***^ *P* < 0.001.

The ADP‐coupled rates of O_2_ consumption in the BF are shown in Fig. 1 *A* for the three respirometry protocols. Total OXPHOS was significantly higher in neonates than at any fetal age and was reduced in TX relative to control fetuses at 127 dGA (Fig. 1 *A*). Pyruvate‐ and PC‐supported oxidative capacity was also higher in newborns than at 104 and 127 dGA (Fig. 1 *A*). Both pyruvate‐ and PC‐supported O_2_ consumption were reduced by TX at 127 and 143 dGA (Fig. 1 *A*).

Leak state respiration, a measure of O_2_ use for processes other than ATP production, differed with age (Table 3). Using malate and either glutamate or pyruvate as substrates, leak state O_2_ consumption increased with increasing age over the perinatal period, with higher rates in newborns than in the younger two groups of fetuses (Table 3). Leak state O_2_ consumption with malate and PC was also higher in newborns than at 127 dGA (Table 3). Leak respiration was not affected by TX at either age using any substrate (Table 3).

**Table 3:** Leak state respiration

	104dGA	127dGA		143dGA		Newborn
	Control	Control	TX	Control	TX	Control
Glutamate	1.12 ± 0.59^a^	0.87 ± 0.28^a^	0.71 ± 0.48	1.47 ± 1.01^a^	0.72 ± 0.30 (*n* = 4)	3.07 ± 1.03^b^
Pyruvate	0.91 ± 0.64^a^	1.29 ± 1.32^a^	0.79 ± 0.36	1.13 ± 0.31^a^	0.70 ± 0.28 (*n* = 4)	3.34 ± 1.81^b^
Palmitoyl carnitine	0.66 ± 0.59^a^	0.39 ± 0.15^a^	0.36 ± 0.15	1.36 ± 0.88^a,b^	0.61 ± 0.28	2.57 ± 1.06 (*n* = 4)^b^

Data are presented as mean ± SD leak state respiration (nmolO_2_ min^–1^ mg^–1^ dry weight). *N* = 5–6 per group unless stated otherwise. Values with different letters are significantly different from each other (*P* < 0.05 by Tukey's *post hoc* test following one‐way ANOVA).

**Figure 2:**
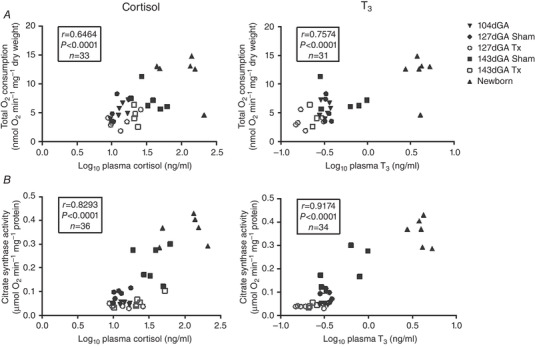
Correlation of biceps femoris mitochondrial parameters with circulating cortisol and T_3_ *A*, ADP‐coupled O_2_ consumption during respiration supported by maximal respiration (malate, glutamate and succinate‐driven; total), and *B*, citrate synthase activity plotted against log_10_ plasma cortisol and T_3_. Data are presented for TX fetuses (open symbols) and controls (filled symbols) at 104 (inverted triangles), 127 (circles) and 143 days of gestational age (dGA; squares) and in newborn lambs (triangles). *r* and *P* values and *n* numbers are given on each graph.

When the data from all the fetal and neonatal groups were combined, irrespective of age or treatment, there were significant positive correlations between the three ADP‐coupled rates of O_2_ consumption and the plasma concentrations of both cortisol and T_3_. These relationships are presented graphically for total OXPHOS in Fig. 2 *A* and as correlation coefficients for the remaining data in Table 4. Because the plasma concentrations of cortisol and T_3_ are also positively related during the perinatal period (*r* = 0.8054, *n* = 34, *P* < 0.0001), partial correlation analyses were carried out using the three factors to identify which hormone concentration had the greater influence on the rates of muscle O_2_ consumption. These analyses showed that T_3_ was statistically the more dominant factor for all three rates of O_2_ consumption as the correlations with T_3_ remained significant when the variation in cortisol levels was taken into account, whereas those with cortisol were not significant in the absence of the confounding effects of the T_3_ concentration (Table 4).

**Table 4:** Correlations, and partial correlation analyses, of plasma hormone concentrations with ADP‐coupled respirometry data from the three protocols, and citrate synthase (CS) and β‐hydroxyacyl‐CoA dehydrogenase (HOAD) activities

	Total	Pyruvate	Palmitoyl carnitine	CS	HOAD
Correlations					
Log_10_plasma cortisol (ng ml^−1^)	*r* = 0.6464 *P* < 0.0001 *n* = 33	*r* = 0.7343 *P* < 0.0001 *n* = 36	*r* = 0.0.5946 *P *= 0.0003 *n* = 32	*r* = 0.8293 *P* < 0.0001 *n* = 36	*r* = 0.8267 *P* < 0.0001 *n* = 36
Log_10_plasma T_3_ (ng ml^−1^)	*r* = 0.7574 *P* < 0.0001 *n* = 31	*r* = 0.8282 *P* < 0.0001 *n* = 34	*r* = 0.7325 *P* < 0.0001 *n* = 30	*r* = 0.9174 *P* < 0.0001 *n* = 34	*r* = 0.8381 *P* < 0.0001 *n* = 34
Partial correlation analyses					
Log_10_plasma cortisol (ng ml^−1^)	*r* = 0.094	*r* = 0.203	*r* = 0.011	***r* = 0.383**	***r* = 0.469**
Log_10_plasma T_3_ (ng ml^−1^)	***r* = 0.523**	***r* = 0.588**	***r* = 0.532**	***r* = 0.753**	***r* = 0.516**

Significant partial correlations are highlighted in bold (*P* < 0.05). *N* = 28–34 for partial correlation analyses.

### Regulating mitochondrial oxidative capacity

#### Metabolic enzyme activities

CS activity, an enzyme of the tricarboxylic acid cycle and a putative marker of mitochondrial density (Larsen *et al*. 2012), increased significantly with age with a two‐fold higher activity at 143 dGA than at the earlier gestational ages and still higher values in neonatal muscle (Fig. 1 *B*). At both 127 and 143 dGA CS activity in the BF was significantly lower in TX than control fetuses (Fig. 1 *B*). Furthermore, the normal increase in CS activity between 127 and 143 dGA was prevented by TX (*P* > 0.05 by *t* test). To determine whether the changes in weight‐specific muscle O_2_ consumption were due to an altered mitochondrial density or functional capacity per unit of mitochondrial mass, the ADP‐coupled rates of O_2_ consumption were normalised to CS activity. There was a trend for decreased ADP‐coupled O_2_ consumption per mitochondrial unit with increasing age irrespective of substrate but this only reached statistical significance with pyruvate and total OXPHOS (Fig. 1 *C*). There was no significant difference in O_2_ consumption per mitochondrial unit in TX fetuses compared to controls at either age using any substrate (Fig. 1 *C*).

The capacity for mitochondrial fat metabolism in the BF was further investigated by measuring the activity of the β‐oxidation enzyme, HOAD. Age had a significant effect on HOAD activity with higher activity at 143 dGA and in the newborn than at 104 dGA (Fig. 1 *D*). Thyroidectomy reduced HOAD activity relative to control values at both ages (Fig. 1 *D*) and prevented the normal ontogenic increase in activity towards term (*P* > 0.05 by *t* test).

When the data from all age groups were combined irrespective of treatment, the activities of CS and HOAD had a significant positive correlation with the plasma concentrations of cortisol and T_3_ (Fig. 2 *B* and Table 4, respectively). Partial correlation analyses showed that both hormones have a significant influence on CS and HOAD activity (Table 4).

#### Mitochondrial ETS proteins

**Figure 3:**
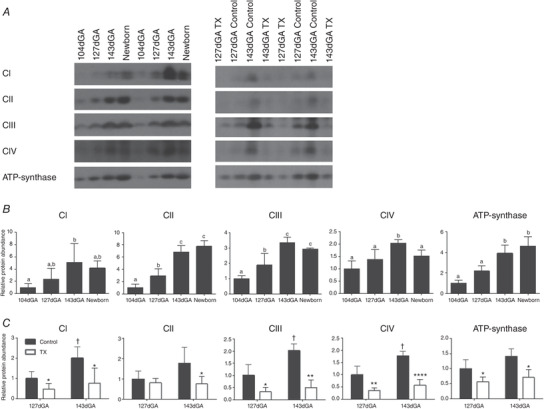
Regulating mitochondrial oxidative capacity in biceps femoris muscle and the impact of thyroidectomy *A*, representative western blots and mean ± SD protein abundance of electron transfer system complexes I–IV (CI–IV) and ATP‐synthase in biceps femoris (*B*) over late gestation and in neonatal muscle and (C) in control (grey bars) and thyroidectomised (TX; open bars) muscle at 127 and 143 dGA. *N* = 5 in each group. Different letters indicate significant difference to each other (*P* < 0.05 by Tukey's *post hoc* test following one‐way ANOVA). Asterisks indicate significant difference from control at the same gestational age: ^*^ *P* < 0.05 (by *t* test); ^**^ *P* < 0.01; ^****^ *P* < 0.0001. ^†^Significantly different from control at 127 dGA: *P* < 0.05 (by *t* test).

Age had a significant influence on protein abundance of ETS complexes I–IV and ATP‐synthase. In general, muscle complex abundance was higher in 143 dGA fetuses and newborns than in the younger fetal groups with no significant differences between the 143 dGA and newborn values in most cases (Fig. 3 *A*). The abundance of several complexes was significantly lower in TX than control fetuses at both ages (Fig. 3 *B*). When there were significant increases in BF complex abundance in controls between 127 and 143dGA, these did not occur in the TX fetuses (Fig. 3 *B*).

#### Mitochondrial regulatory genes

**Figure 4:**
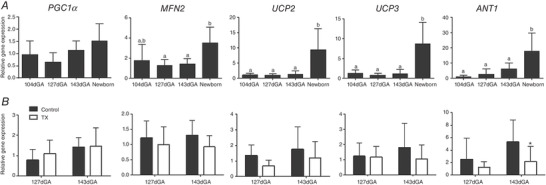
Regulating mitochondrial oxidative capacity in biceps femoris muscle and the impact of thyroidectomy Values are mean ± SD mRNA levels of peroxisome proliferator‐activated receptor gamma coactivator 1 alpha (PGC1α), mitofusin 2 (MFN2), uncoupling proteins (UCP) 2 and 3, and adenine nucleotide translocase 1 (ANT1): *A*, over late gestation and in neonatal muscle; and *B*, in control (grey bars) and thyroidectomised (TX; open bars) muscle at 127 and 143 dGA. *N* = 5–6 in each group. Different letters indicate significant difference to each other (*P* < 0.05 by Tukey's *post hoc* test following one‐way ANOVA). ^*^Significantly different from control at the same gestational age (*P* < 0.05 by *t* test).

The expression of genes encoding PGC1α and MFN2 were measured as regulators of mitochondrial biogenesis and fusion, respectively. *MFN2* but not *PGC1α* expression was influenced by age (Fig. 4 *A*). Expression of neither gene was affected by TX (Fig. 4 *B*). Expression of IMM proteins known to dissipate the proton gradient were also quantified. Both *UCP2* and *UCP3* gene expression were upregulated 5‐ to 7‐fold in the newborn compared with all fetal groups (Fig. 4 *A*) but were not affected statistically by TX (Fig. 4 *B*). Expression of the *ANT1* gene was significantly higher in newborns than in all fetus groups (Fig. 4 *A*) and was lower in TX than control fetuses at 143 dGA (Fig. 4 *B*).

#### Muscle morphology

**Figure 5:**
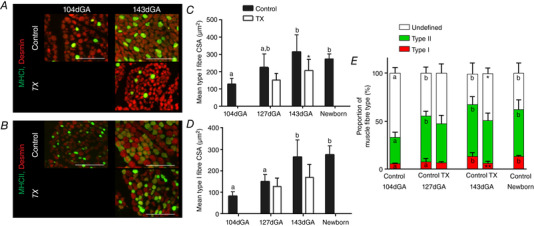
Maturation of late gestation and neonatal biceps femoris muscle structure and the impact of thyroidectomy *A* and *B*, immunostaining of type I (*A*) and type II (*B*) fibres; representative 10 μm cross sections stained for myosin heavy chain isoforms I and II (green) and counterstained for desmin (red) at 104 and 143 days of gestational age (dGA) as labelled. Muscle sections from sham and thyroidectomised (TX) fetuses are shown at 143 dGA. White scale bar = 100 μm. *C* and *D*, mean ± SD cross sectional area (CSA) of type I (*C*) and type II (*D*) fibres. Control data are shown in grey and thyroidectomy (TX) data in open bars. *E*, mean ± SD proportion of type I (red), type II (green) and undefined (white) fibres of fetuses at 104, 127 (control and TX) and 143 dGA (control and TX) and newborn lambs. *N* = 5–6 in each group. Different letters indicate significant difference to each other (*P* < 0.05 by Tukey's *post hoc* test following one‐way ANOVA). Asterisks indicate significant difference from control at the same gestational age: ^*^ *P* < 0.05 (by *t* test); ^**^ *P* < 0.01.

**Figure 6:**
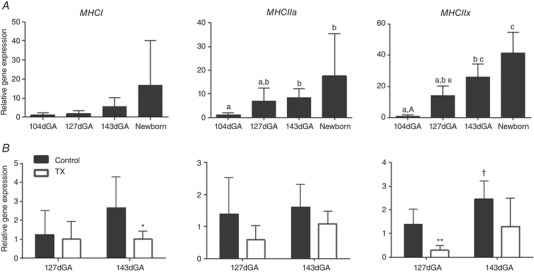
Maturation of fetal and neonatal skeletal muscle structure and the impact of thyroidectomy Mean ± SD gene expression of MHCI, MHCIIa and MHCIIx in fetuses at 104, 127 and 143 days of gestational age (dGA) and newborn lambs (*A*) and 127 dGA and 143 dGA sham (grey) and thyroidectomised (TX; open bars) fetuses (*B*) in biceps femoris. *N* = 4–6 per group. Values with different letters are significantly different from each other (*P* < 0.05 by Tukey's post hoc test following one‐way ANOVA). Asterisks indicate significant difference from control at the same gestational age: ^*^ *P* < 0.05 (by *t* test); ^**^ *P* < 0.01). ^†^Significantly different from control at 127 dGA: *P* < 0.05 (by *t* test).

Age had a significant positive effect on both the cross‐sectional area (CSA) and proportions of type I and II fibres in the BF (Fig. 5). In addition, gene expression of MHCI, IIa and IIx showed a significant positive effect of age on *MHCIIa* and *MHCIIx* (Fig. 6 *A*). The proportion and CSA of type I fibres were reduced in BF of TX fetuses relative to controls at 143 dGA (Fig. 5 *B*). Thyroidectomy reduced expression of *MHCIIx* at 127 dGA and *MHCI* at 143 dGA relative to control values (Fig. 6 *B*). In addition, TX prevented the normal ontogenic increase in *MHCIIx* seen in the controls between 127 and 143 dGA (*P* > 0.05 by *t* test in all cases).

## Discussion

This study demonstrated that, in ovine BF skeletal muscle, mitochondrial oxidative capacity increased significantly with age in a substrate‐dependent manner, with the rate of O_2_ consumption higher in neonatal than fetal muscle particularly when using carbohydrates. Over late gestation there was an increase in mitochondrial density and abundance of ETS and other proteins involved in oxidative ATP generation, consistent with other prepartum maturational processes known to ensure a successful transition to extrauterine life (Fowden *et al*. 1998). Fetal hypothyroidism induced by surgical removal of the fetal thyroid glands showed that thyroid hormones are necessary for most of the developmental changes in mitochondrial function that occur towards term. The BF from TX fetuses had a significantly lower mitochondrial density and abundance of proteins involved in oxidative ATP production near term, as well as delayed maturation of its muscle fibre composition. Indeed, when the data from all the animals were combined, there were significant positive correlations between the oxidative capacity of the BF and the circulating concentrations of cortisol and T_3_, both of which normally act as signals for maturation and of proximity to delivery. Because cortisol controls the deiodination of T_4_ to T_3_ close to term (Forhead & Fowden, 2014), the current findings suggest that these hormones act cooperatively in regulating mitochondrial function over late gestation, as has been shown previously for other aspects of prepartum maturation (Forhead *et al*. 2002, 1998).

### Development of mitochondrial oxidative capacity over the perinatal period

Respiration in the presence of saturating concentrations of malate, glutamate, succinate and ADP indicated an age‐dependent rise in electron transport chain capacity that was dependent on thyroid hormones. To investigate the mitochondrial capacity to oxidise more physiological substrates, we measured respiration in the presence of saturating concentrations of malate plus pyruvate and, separately, malate plus PC. We found that ovine fetal skeletal muscle mitochondria have the capacity to use both carbohydrate‐derived (i.e. pyruvate) and fatty acid‐derived (i.e. PC) substrates for oxidative metabolism throughout the last third of gestation. In normal conditions, *in vivo* studies of the whole sheep fetus show that carbohydrate in the form of glucose is its predominant metabolic substrate although free fatty acids are available in the fetal circulation during late gestation and rise rapidly in concentration postnatally (James *et al*. 1971; Mellor & Cockburn, 1986). Seemingly, in line with these findings, the current results show that rates of O_2_ consumption using pyruvate were around 2–3 times higher than those seen with PC but, in mitochondrial preparations *in vitro*, pyruvate typically supports higher respiration rates than fatty acid‐derived substrates, due to more rapid production of reducing intermediates. Notably, there was a perinatal increase in muscle activity of the fatty acid β‐oxidation enzyme, HOAD, in the current study, which indicates that fat may become a more prominent metabolic substrate postnatally with the onset of a milk diet with its high fat content (Herrara & Amusquivar, 2000).

The major increase in muscle O_2_ consumption occurred neonatally with little, if any, change during late gestation, consistent with previous findings of a 58% increase in the whole body rate of O_2_ consumption in the newborn lamb (Smolich *et al*. 1992). However, mitochondrial density, as measured by CS activity, and the abundance of ETS proteins increased in late gestation in advance of delivery. As there was no significant change in gene expression of *PGC1α* before birth, *PGC1α* is unlikely to be a major regulatory factor of mitochondrial density over late gestation. The increased mitochondrial density may relate to the increased proportion of type I fibres that have more mitochondria over this period of development. There was also an increase in *MFN2* expression postnatally compared with 145 dGA, indicating increased mitochondrial fusion, which, in turn, is associated with enhanced ATP production when energy demands are high (Schrepfer & Scorrano, 2016). The positive correlations observed between the cortisol concentration and the activities of CS and HOAD, as well as with the rates of O_2_ consumption, suggest that prepartum maturation of mitochondrial oxidative capacity may be dependent on cortisol, as occurs with other metabolic processes towards term (Fowden *et al*. 1998). Collectively, our findings show that there is prepartum upregulation of capacity for mitochondrial oxidative phosphorylation in anticipation of the increased neonatal ATP requirement.

The temporal discrepancy between the increments in mitochondrial density and total OXPHOS capacity is a novel finding and indicates that different regulatory mechanisms may drive prenatal maturation and neonatal upregulation of mitochondrial oxidative function in skeletal muscle. *In vivo*, neonatal upregulation of mitochondrial O_2_ consumption capacity may be due to either removal of an inhibitory feto‐placental factor or to a positive stimulus associated with delivery and/or the extra‐uterine environment, for example due to alterations in the electrolyte, O_2_ and substrate availability (Ogbi & Johnson, 2006; Huttemann *et al*. 2007; Solaini *et al*. 2010). In the current *in vitro* experimental setting, however, these factors are controlled and, thus, altered expression of mitochondrial factors, such as the observed upregulation of *ANT1*, may play a crucial regulatory role in the postnatal increase in OXPHOS and O_2_ consumption by maintaining ADP levels in the mitochondrial matrix to support ATP‐synthase activity. The delay in upregulating mitochondrial respiratory capacity until after delivery, despite the prepartum increase in mitochondrial density, may be beneficial in restricting fetal O_2_ demand *in utero* when the O_2_ supply is limited.

The marked neonatal increase in *UCP* and *ANT1* expression will also contribute to minimising superoxide production by dissipating the proton gradient across the IMM (Arsenijevic *et al*. 2000; Vidal‐Puig *et al*. 2000; Brand *et al*. 2005), in line with the ontogenic increase in leak state respiration observed perinatally in the current study. Although proton leak is thought to reduce the efficiency of oxidative phosphorylation, concomitant increases in ATP synthesis and UCP expression have been reported previously in rats, suggesting that uncoupling may have a negligible effect on ATP availability when oxidative phosphorylation is working at maximal rates (Vidal‐Puig *et al*. 2000; Short *et al*. 2001). Muscle *UCP3* may also be involved in upregulating lipid metabolism after birth (Clapham *et al*. 2000). In addition, the 2‐ to 3‐fold increment in *ANT1* abundance after birth may increase both mitochondrial availability of ADP required for OXPHOS and the cellular availability of ATP, by enhancing transport of ADP into, and ATP out of, the inner mitochondrial space (Brand *et al*. 2005).

### Developmental effects of fetal hypothyroidism

As in postnatal tissue (Lombardi *et al*. 2015), the current study shows that thyroid hormones are important metabolic regulators in fetal skeletal muscle. In late gestation, O_2_ consumption by TX muscles was lower than control values in a substrate‐specific manner, consistent with the low whole body rates of O_2_ consumption in TX sheep fetuses (Fowden & Silver, 1995). The lower rates of muscle O_2_ consumption after TX were accompanied by reductions in CS activity and the abundance of ETS complexes and ATP‐synthase. These decrements were more pronounced at 143 than at 127 dGA. TX muscles also had lower HOAD activity and expression of *ANT1* than control muscles by 143 dGA. In addition, TX abolished the developmental increase in mitochondrial oxidative capacity seen in control muscle between 127 and 143 dGA. Furthermore, TX was associated with a lower proportion of type I fibres and more undifferentiated muscle fibres in the BF at 143 dGA, in line with previous studies of other skeletal muscles in fetal sheep (Finkelstein *et al*. 1991). Together, the current findings suggest that the effects of thyroid hormones on muscle mitochondrial function *in utero* may be mediated at least, in part, by their role in muscle fibre differentiation.

Thyroid hormones may act directly to influence fetal muscle fibre type and mitochondrial function as their receptors are known to be expressed in fetal skeletal muscle during late gestation (Duchamp *et al*. 1994). The rise in the proportion of type II fibres between 105 and 127 dGA, despite no alteration in cortisol or T_3_ concentrations, suggests that changes in the abundance of their hormone receptors or the tissue deiodinases may contribute to the normal developmental changes in skeletal muscle, as occurs in other fetal tissues over this period of gestation (Polk *et al*. 1989; Mostyn *et al*. 2004; Wu *et al*. 2005). Alternatively, the actions of thyroid hormones may be indirect and mediated through other hormones or fetal tissues, which then affect muscle development. Previous studies in fetal sheep have shown that thyroid hormone deficiency affects bioavailability of insulin and insulin‐like growth factors, both of which are known to affect muscle development *in utero* (Forhead *et al*. 2002; Harris *et al*. 2017). Fetal hypothyroidism also impairs normal cerebral development, which in turn may reduce neuronal stimulation of skeletal muscle with known developmental effects on the proportion of different fibre types in rats (Gambke *et al*. 1983; Horn & Heuer, 2010). Whatever the mechanism involved *in utero*, the positive correlations observed between the fetal T_3_ concentrations and both muscle O_2_ consumption and mitochondrial density show that fetal thyroid hormones have an important role in controlling mitochondrial oxidative capacity during the immediate prepartum period, consistent with the role of these hormones in increasing metabolic rate in the adult (Harper & Seifert, 2008). Indeed, partial correlation analyses suggest T_3_ is the dominant factor driving development of mitochondrial function, albeit this may be a T_3_‐mediated, cortisol‐dependent process (Forhead & Fowden, 2014). However, further studies are needed using adrenalectomised fetuses and T_4_/T_3_ replacement in TX fetuses to establish the precise nature of this endocrine interdependence.

## Conclusion

Collectively, the results show that the capacity of skeletal muscle for ATP production increases *in utero* in advance of the extra energy requirement at birth by increasing the number of mitochondria and their functional capacity in a hormone‐dependent manner. However, there is little evidence for any increase in the rate of O_2_ consumption *per se* until after delivery. Coupled with the decline in oxidative capacity per mitochondrial unit, this may be a mechanism to more effectively control superoxide production and oxidative stress during the transition from intra‐ to extra‐uterine life. The thyroid hormones appear to influence mitochondrial oxidative capacity via effects on myogenesis and differentiation of the muscle fibres while other factors, such as the fetal cortisol concentration, enhance mitochondrial biogenesis and ETS complex abundance in differentiated fibres close to term. However, further studies are required to identify the specific molecular mechanisms and signalling pathways involved in the prepartum maturation and neonatal upregulation of mitochondrial OXPHOS. A greater understanding of these regulatory processes and their endocrine dependence *in utero* is likely to have beneficial consequences for the viability and long‐term metabolic health of infants born pre‐term or with prenatal thyroid hormone deficiency.

## Additional information

### Competing interests

The authors declare no competing interests.

### Author contributions

Conceptualisation, methodology, funding acquisition and writing – the study was designed and funded by grant applications by K.L.D., A.J.M. and A.L.F. The experimental work on the animals was carried out by K.L.D., A.J.F., E.J.C. and A.L.F. The tissue analyses were carried out by K.L.D., E.J.C., A.J.M., E.V.A. and T.L. The manuscript was written by K.L.D., A.L.F. and A.J.M. and commented on by E.J.C. and A.J.F.

### Funding

This work was supported by the Wellcome Trust and by Biotechnology and Biological Sciences Research Council (BB/P019048/1). K.L.D. was supported, in part, by a Wellcome Trust PhD studentship (grant code 102357/Z/13/A).

## Supporting information


**Statistical Summary Document**
