# Supplementary material for: Development and thyroid hormone dependence of skeletal muscle mitochondrial function towards birth
Source: J Physiol. 2020 Mar 27;598(12):2453–68. doi: 10.1113/JP279194 (PMC7317365; doi:10.1113/JP279194)
Supplement: Supplementary file 1 — Statistical Summary Document [file TJP-598-2453-s001.docx]

**Manuscript Title: Development and thyroid hormone dependence of skeletal muscle mitochondrial function towards birth**

**Authors:** K. L. Davies^1^*, E. J. Camm^1^, E. V. Atkinson^1^, T. Lopez^1^, A. J. Forhead^1,2^, A. J. Murray^1^, A. L. Fowden^1^

**Animal model used, if applicable:** Welsh mountain sheep

**Underlying hypothesis:**

We hypothesise that there may be thyroid hormone-dependent changes in mitochondrial abundance and function over late gestation in preparation for birth.

**Definitions of ‘n’:**

Number of samples (one sample per fetus/lamb)

**Statistical summary table:**

| Experimental question number* | Finding/ conclusion | Experimental location/ variable  e.g. cortex vs cerebellum or genotype | Mean value  (or other summary statistic) | SD | n (value) | P** | Units | Data comparisons  e.g. WT vs KO | Statistical test | Any other variable  e.g. subjects’ age or sex | Figure/table in which data are presented | Comments  e.g. observation |
| --- | --- | --- | --- | --- | --- | --- | --- | --- | --- | --- | --- | --- |
| 1. Total oxygen consumption | Increases with age; thyroid dependent | 104 days of gestational age (dGA) | 5.51 | 1.27 | 6 | **0.0008** | nmol O2/min/mg dry weight | Effect of age across the 4 age groups | One-way ANOVA |  | Figure 1 |  |
|  |  | 127dGA | 5.80 | 2.08 | 6 |  |  |  |  |  |  |  |
|  |  | 143dGA | 7.34 | 2.07 | 6 |  |  |  |  |  |  |  |
|  |  | Newborn | 11.84 | 3.62 | 6 |  |  |  |  |  |  |  |
|  |  | 127dGA thyroidectomised (TX) | 3.55 | 1.24 | 6 | **0.0462** |  | TX vs age-matched control (AMC) | Student’s t-test, unpaired (t-test) |  |  |  |
|  |  | 143dGA TX | 4.82 | 1.59 | 5 | 0.0667 |  | TX vs AMC | t-test (non-parametric; NP) |  |  |  |
| 2. Pyruvate-supported oxygen consumption | Increases with age; thyroid dependent | 104dGA | 5.00 | 1.69 | 6 | **<0.0001** | nmol O2/min/mg dry weight | Effect of age across the 4 age groups | One-way ANOVA |  | Figure 1 |  |
|  |  | 127dGA | 6.66 | 2.44 | 6 |  |  |  |  |  |  |  |
|  |  | 143dGA | 7.71 | 1.36 | 6 |  |  |  |  |  |  |  |
|  |  | Newborn | 13.84 | 3.77 | 6 |  |  |  |  |  |  |  |
|  |  | 127dGA TX | 4.01 | 0.92 | 6 | **0.0320** |  | TX vs AMC | t-test |  |  |  |
|  |  | 143dGA TX | 4.41 | 1.30 | 6 | **0.0016** |  | TX vs AMC | t-test |  |  |  |
| 3. Palmitoyl carnitine-supported oxygen consumption | Increases with age; thyroid dependent | 104dGA | 1.60 | 0.61 | 5 | **0.0038** | nmol O2/min/mg dry weight | Effect of age across the 4 age groups | One-way ANOVA |  | Figure 1 |  |
|  |  | 127dGA | 1.99 | 0.97 | 6 |  |  |  |  |  |  |  |
|  |  | 143dGA | 2.87 | 0.83 | 5 |  |  |  |  |  |  |  |
|  |  | Newborn | 5.48 | 2.75 | 4 |  |  |  |  |  |  |  |
|  |  | 127dGA TX | 1.13 | 0.54 | 6 | **0.0411** |  | TX vs AMC | t-test (NP) |  |  |  |
|  |  | 143dGA TX | 1.20 | 0.51 | 6 | **0.0043** |  | TX vs AMC | t-test (NP) |  |  |  |
| 4. Citrate synthase activity | Increases with age; thyroid dependent | 104dGA | 0.050 | 0.005 | 6 | **<0.0001** | µmol/min/mg protein | Effect of age across the 4 age groups | One-way ANOVA |  | Figure 1 |  |
|  |  | 127dGA | 0.089 | 0.023 | 6 |  |  |  |  |  |  |  |
|  |  | 143dGA | 0.219 | 0.074 | 6 |  |  |  |  |  |  |  |
|  |  | Newborn | 0.359 | 0.059 | 6 |  |  |  |  |  |  |  |
|  |  | 127dGA TX | 0.039 | 0.007 | 6 | **0.0005** |  | TX vs AMC | t-test |  |  |  |
|  |  | 143dGA TX | 0.057 | 0.026 | 6 | **0.0005** |  | TX vs AMC | t-test |  |  |  |
| 5. HOAD activity | Increases with age; thyroid dependent | 104dGA | 0.320 | 0.032 | 6 | **<0.0001** | µmol/min/mg protein | Effect of age across the 4 age groups | One-way ANOVA |  | Figure 1 |  |
|  |  | 127dGA | 0.552 | 0.145 | 6 |  |  |  |  |  |  |  |
|  |  | 143dGA | 1.080 | 0.239 | 6 |  |  |  |  |  |  |  |
|  |  | Newborn | 1.238 | 0.150 | 6 |  |  |  |  |  |  |  |
|  |  | 127dGA TX | 0.317 | 0.054 | 6 | **0.0040** |  | TX vs AMC | t-test |  |  |  |
|  |  | 143dGA TX | 0.435 | 0.127 | 6 | **0.0002** |  | TX vs AMC | t-test |  |  |  |
| 6. Relative protein abundance of complex I | Increases with age; thyroid dependent | 104dGA | 1.00 | 0.67 | 5 | **0.0128** | Relative protein abundance | Effect of age across the 4 age groups | One-way ANOVA |  | Figure 3 |  |
|  |  | 127dGA | 2.34 | 1.78 | 5 |  |  |  |  |  |  |  |
|  |  | 143dGA | 5.12 | 2.98 | 5 |  |  |  |  |  |  |  |
|  |  | Newborn | 4.18 | 1.14 | 5 |  |  |  |  |  |  |  |
|  |  | 127dGA control | 1.00 | 0.34 | 5 | **0.0280** |  | TX vs AMC | t-test |  |  |  |
|  |  | 127dGA TX | 0.47 | 0.28 | 5 |  |  |  |  |  |  |  |
|  |  | 143dGA control | 2.01 | 0.57 | 5 | **0.0181** |  | TX vs AMC | t-test |  |  |  |
|  |  | 143dGA TX | 0.75 | 0.75 | 5 |  |  |  |  |  |  |  |
| 7. Relative protein abundance of complex II | Increases with age; impact of TX at 143dGA | 104dGA | 1.00 | 0.57 | 5 | **<0.0001** | Relative protein abundance | Effect of age across the 4 age groups | One-way ANOVA |  | Figure 3 |  |
|  |  | 127dGA | 2.92 | 1.14 | 5 |  |  |  |  |  |  |  |
|  |  | 143dGA | 6.86 | 0.98 | 5 |  |  |  |  |  |  |  |
|  |  | Newborn | 7.77 | 0.95 | 5 |  |  |  |  |  |  |  |
|  |  | 127dGA control | 1.00 | 0.38 | 5 | 0.3895 |  | TX vs AMC | t-test |  |  |  |
|  |  | 127dGA TX | 0.82 | 0.21 | 5 |  |  |  |  |  |  |  |
|  |  | 143dGA control | 1.77 | 0.79 | 5 | **0.0312** |  | TX vs AMC | t-test |  |  |  |
|  |  | 143dGA TX | 0.76 | 0.35 | 5 |  |  |  |  |  |  |  |
| 8. Relative protein abundance of complex III | Increases with age; thyroid dependent | 104dGA | 1.00 | 0.19 | 5 | **<0.0001** | Relative protein abundance | Effect of age across the 4 age groups | One-way ANOVA |  | Figure 3 |  |
|  |  | 127dGA | 1.89 | 0.75 | 5 |  |  |  |  |  |  |  |
|  |  | 143dGA | 3.35 | 0.34 | 5 |  |  |  |  |  |  |  |
|  |  | Newborn | 2.93 | 0.07 | 5 |  |  |  |  |  |  |  |
|  |  | 127dGA control | 1.00 | 0.44 | 5 | **0.0134** |  | TX vs AMC | t-test |  |  |  |
|  |  | 127dGA TX | 0.33 | 0.17 | 5 |  |  |  |  |  |  |  |
|  |  | 143dGA control | 2.04 | 0.27 | 5 | **0.0079** |  | TX vs AMC | t-test (NP) |  |  |  |
|  |  | 143dGA TX | 0.50 | 0.30 | 5 |  |  |  |  |  |  |  |
| 9. Relative protein abundance of complex IV | Increases with age; thyroid dependent | 104dGA | 1.00 | 0.32 | 5 | **0.0004** | Relative protein abundance | Effect of age across the 4 age groups | One-way ANOVA |  | Figure 3 |  |
|  |  | 127dGA | 1.38 | 0.39 | 5 |  |  |  |  |  |  |  |
|  |  | 143dGA | 2.04 | 0.15 | 5 |  |  |  |  |  |  |  |
|  |  | Newborn | 1.52 | 0.26 | 5 |  |  |  |  |  |  |  |
|  |  | 127dGA control | 1.00 | 0.35 | 5 | **0.0079** |  | TX vs AMC | t-test (NP) |  |  |  |
|  |  | 127dGA TX | 0.35 | 0.11 | 5 |  |  |  |  |  |  |  |
|  |  | 143dGA control | 1.78 | 0.18 | 5 | **<0.0001** |  | TX vs AMC | t-test |  |  |  |
|  |  | 143dGA TX | 0.57 | 0.24 | 5 |  |  |  |  |  |  |  |
| 10. Relative protein abundance of ATP-synthase | Increases with age; thyroid dependent | 104dGA | 1.00 | 0.30 | 5 | **<0.0001** | Relative protein abundance | Effect of age across the 4 age groups | One-way ANOVA |  | Figure 3 |  |
|  |  | 127dGA | 2.20 | 0.51 | 5 |  |  |  |  |  |  |  |
|  |  | 143dGA | 3.93 | 0.80 | 5 |  |  |  |  |  |  |  |
|  |  | Newborn | 4.61 | 0.92 | 5 |  |  |  |  |  |  |  |
|  |  | 127dGA control | 1.00 | 0.30 | 5 | **0.0204** |  | TX vs AMC | t-test |  |  |  |
|  |  | 127dGA TX | 0.56 | 0.16 | 5 |  |  |  |  |  |  |  |
|  |  | 143dGA control | 1.41 | 0.25 | 5 | **0.0159** |  | TX vs AMC | t-test |  |  |  |
|  |  | 143dGA TX | 0.71 | 0.26 | 5 |  |  |  |  |  |  |  |
| 11. mRNA levels of PGC1a | Not significantly affected by age or TX | 104dGA | 0.95 | 0.56 | 5 | 0.0783 | Relative gene expression | Effect of age across the 4 age groups | One-way ANOVA |  | Figure 4 |  |
|  |  | 127dGA | 0.66 | 0.37 | 6 |  |  |  |  |  |  |  |
|  |  | 143dGA | 1.14 | 0.38 | 6 |  |  |  |  |  |  |  |
|  |  | Newborn | 1.51 | 0.70 | 5 |  |  |  |  |  |  |  |
|  |  | 127dGA control | 0.79 | 0.51 | 5 | 0.4201 |  | TX vs AMC | t-test |  |  |  |
|  |  | 127dGA TX | 1.10 | 0.67 | 6 |  |  |  |  |  |  |  |
|  |  | 143dGA control | 1.42 | 0.48 | 6 | 0.9164 |  | TX vs AMC | t-test |  |  |  |
|  |  | 143dGA TX | 1.47 | 0.92 | 6 |  |  |  |  |  |  |  |
| 12. mRNA levels of MFN2 | Increases with age; not significantly affected by TX | 104dGA | 1.77 | 1.57 | 5 | **0.0124** | Relative gene expression | Effect of age across the 4 age groups | One-way ANOVA |  | Figure 4 |  |
|  |  | 127dGA | 1.29 | 0.55 | 6 |  |  |  |  |  |  |  |
|  |  | 143dGA | 1.42 | 0.22 | 6 |  |  |  |  |  |  |  |
|  |  | Newborn | 3.51 | 0.65 | 6 |  |  |  |  |  |  |  |
|  |  | 127dGA control | 1.22 | 0.56 | 5 | 0.5400 |  | TX vs AMC | t-test |  |  |  |
|  |  | 127dGA TX | 1.00 | 0.59 | 6 |  |  |  |  |  |  |  |
|  |  | 143dGA control | 1.30 | 0.20 | 6 | 0.1602 |  | TX vs AMC | t-test |  |  |  |
|  |  | 143dGA TX | 0.92 | 0.37 | 6 |  |  |  |  |  |  |  |
| 13. mRNA levels of UCP2 | Increases with age; not significantly affected by TX | 104dGA | 1.02 | 0.50 | 5 | **0.0014** | Relative gene expression | Effect of age across the 4 age groups | One-way ANOVA |  | Figure 4 |  |
|  |  | 127dGA | 0.95 | 0.45 | 6 |  |  |  |  |  |  |  |
|  |  | 143dGA | 1.28 | 1.05 | 6 |  |  |  |  |  |  |  |
|  |  | Newborn | 9.36 | 6.93 | 6 |  |  |  |  |  |  |  |
|  |  | 127dGA control | 1.36 | 0.68 | 5 | 0.0599 |  | TX vs AMC | t-test |  |  |  |
|  |  | 127dGA TX | 0.68 | 0.36 | 6 |  |  |  |  |  |  |  |
|  |  | 143dGA control | 1.75 | 0.59 | 6 | 0.4592 |  | TX vs AMC | t-test |  |  |  |
|  |  | 143dGA TX | 1.19 | 1.04 | 6 |  |  |  |  |  |  |  |
| 14. mRNA levels of UCP3 | Increases with age; not significantly affected by TX | 104dGA | 1.25 | 0.86 | 5 | **0.0002** | Relative gene expression | Effect of age across the 4 age groups | One-way ANOVA |  | Figure 4 |  |
|  |  | 127dGA | 0.78 | 0.50 | 6 |  |  |  |  |  |  |  |
|  |  | 143dGA | 1.18 | 1.05 | 6 |  |  |  |  |  |  |  |
|  |  | Newborn | 8.74 | 5.39 | 6 |  |  |  |  |  |  |  |
|  |  | 127dGA control | 1.23 | 0.86 | 5 | 0.9110 |  | TX vs AMC | t-test |  |  |  |
|  |  | 127dGA TX | 1.18 | 0.69 | 6 |  |  |  |  |  |  |  |
|  |  | 143dGA control | 1.80 | 1.60 | 6 | 0.3446 |  | TX vs AMC | t-test |  |  |  |
|  |  | 143dGA TX | 1.06 | 0.91 | 6 |  |  |  |  |  |  |  |
| 15. mRNA levels of ANT1 | Increases with age; significantly lower in TX fetuses than controls at 143dGA | 104dGA | 0.97 | 0.86 | 5 | **0.0018** | Relative gene expression | Effect of age across the 4 age groups | One-way ANOVA |  | Figure 4 |  |
|  |  | 127dGA | 2.58 | 1.44 | 6 |  |  |  |  |  |  |  |
|  |  | 143dGA | 6.05 | 1.66 | 6 |  |  |  |  |  |  |  |
|  |  | Newborn | 17.86 | 4.84 | 5 |  |  |  |  |  |  |  |
|  |  | 127dGA control | 2.53 | 3.39 | 5 | 0.4017 |  | TX vs AMC | t-test |  |  |  |
|  |  | 127dGA TX | 1.27 | 0.87 | 6 |  |  |  |  |  |  |  |
|  |  | 143dGA control | 5.32 | 3.57 | 6 | **0.0476** |  | TX vs AMC | t-test (NP) |  |  |  |
|  |  | 143dGA TX | 2.17 | 2.41 | 6 |  |  |  |  |  |  |  |
| 16. mRNA levels of MHCI | Not significantly affected by age; significantly lower in TX fetuses than controls at 143dGA | 104dGA | 1.18 | 1.02 | 5 | 0.1522 | Relative gene expression | Effect of age across the 4 age groups | One-way ANOVA |  | Figure 6 |  |
|  |  | 127dGA | 1.80 | 1.73 | 6 |  |  |  |  |  |  |  |
|  |  | 143dGA | 5.61 | 4.54 | 6 |  |  |  |  |  |  |  |
|  |  | Newborn | 16.65 | 23.47 | 6 |  |  |  |  |  |  |  |
|  |  | 127dGA control | 1.24 | 1.28 | 5 | 0.6494 |  | TX vs AMC | t-test (NP) |  |  |  |
|  |  | 127dGA TX | 1.02 | 0.91 | 6 |  |  |  |  |  |  |  |
|  |  | 143dGA control | 2.66 | 1.66 | 5 | **0.0425** |  | TX vs AMC | t-test |  |  |  |
|  |  | 143dGA TX | 1.02 | 0.43 | 6 |  |  |  |  |  |  |  |
| 17. mRNA levels of MHCIIa | Not significantly affected by age or TX | 104dGA | 1.11 | 0.81 | 5 | 0.0071 | Relative gene expression | Effect of age across the 4 age groups | One-way ANOVA (NP) |  | Figure 6 |  |
|  |  | 127dGA | 6.95 | 5.36 | 6 |  |  |  |  |  |  |  |
|  |  | 143dGA | 8.41 | 3.73 | 6 |  |  |  |  |  |  |  |
|  |  | Newborn | 17.62 | 17.96 | 4 |  |  |  |  |  |  |  |
|  |  | 127dGA control | 1.39 | 1.13 | 5 | 0.1255 |  | TX vs AMC | t-test (NP) |  |  |  |
|  |  | 127dGA TX | 0.59 | 0.43 | 6 |  |  |  |  |  |  |  |
|  |  | 143dGA control | 1.61 | 0.71 | 6 | 0.1464 |  | TX vs AMC | t-test |  |  |  |
|  |  | 143dGA TX | 1.08 | 0.39 | 6 |  |  |  |  |  |  |  |
| 18. mRNA levels of MHCIIx | Increases with age; significantly lower in TX fetuses than controls at 127dGA | 104dGA | 1.03 | 0.66 | 5 | **<0.0001** | Relative gene expression | Effect of age across the 4 age groups | One-way ANOVA |  | Figure 6 |  |
|  |  | 127dGA | 13.99 | 6.51 | 6 |  |  |  |  |  |  |  |
|  |  | 143dGA | 26.07 | 8.23 | 6 |  |  |  |  |  |  |  |
|  |  | Newborn | 41.42 | 13.46 | 5 |  |  |  |  |  |  |  |
|  |  | 127dGA control | 1.38 | 0.66 | 5 | **0.0039** |  | TX vs AMC | t-test |  |  |  |
|  |  | 127dGA TX | 0.30 | 0.19 | 6 |  |  |  |  |  |  |  |
|  |  | 143dGA control | 2.45 | 0.78 | 6 | 0.0740 |  | TX vs AMC | t-test |  |  |  |
|  |  | 143dGA TX | 1.29 | 1.20 | 6 |  |  |  |  |  |  |  |
| 19. Mean type I fibre CSA | Increases with age; significantly lower in TX fetuses than controls at 143dGA | 104dGA | 126.8 | 34.6 | 6 | **0.0008** | µm2 | Effect of age across the 4 age groups | One-way ANOVA |  | Figure 5 |  |
|  |  | 127dGA | 224.3 | 77.0 | 6 |  |  |  |  |  |  |  |
|  |  | 143dGA | 315.0 | 96.4 | 6 |  |  |  |  |  |  |  |
|  |  | Newborn | 272.5 | 30.7 | 5 |  |  |  |  |  |  |  |
|  |  | 127dGA TX | 150.4 | 38.6 | 6 | 0.0649 |  | TX vs AMC | t-test (NP) |  |  |  |
|  |  | 143dGA TX | 206.3 | 64.3 | 6 | **0.0445** |  | TX vs AMC | t-test |  |  |  |
| 20. Type II CSA | Increases with age; unaffected by TX | 104dGA | 82.5 | 20.0 | 6 | **<0.0001** | µm2 | Effect of age across the 4 age groups | One-way ANOVA |  | Figure 5 |  |
|  |  | 127dGA | 150.9 | 30.8 | 6 |  |  |  |  |  |  |  |
|  |  | 143dGA | 265.1 | 78.9 | 5 |  |  |  |  |  |  |  |
|  |  | Newborn | 275.5 | 41.6 | 5 |  |  |  |  |  |  |  |
|  |  | 127dGA TX | 127.7 | 38.0 | 6 | 0.2717 |  | TX vs AMC | t-test |  |  |  |
|  |  | 143dGA TX | 169.4 | 59.6 | 5 | 0.0625 |  | TX vs AMC | t-test |  |  |  |
| 21. Percentage of area accounted for by type I fibres | Increases with age; significantly lower in TX fetuses than controls at 143dGA | 104dGA | 5.57 | 0.85 | 6 | **0.0004** | % | Effect of age across the 4 age groups | One-way ANOVA |  | Figure 5 |  |
|  |  | 127dGA | 7.36 | 3.72 | 6 |  |  |  |  |  |  |  |
|  |  | 143dGA | 12.93 | 4.19 | 6 |  |  |  |  |  |  |  |
|  |  | Newborn | 13.18 | 1.07 | 5 |  |  |  |  |  |  |  |
|  |  | 127dGA TX | 6.45 | 1.35 | 6 | 0.5842 |  | TX vs AMC | t-test |  |  |  |
|  |  | 143dGA TX | 5.90 | 2.30 | 6 | **0.0049** |  | TX vs AMC | t-test |  |  |  |
| 22. Percentage of area accounted for by type II fibres | Increases with age; not significantly affected by TX | 104dGA | 27.28 | 5.62 | 6 | **<0.0001** | % | Effect of age across the 4 age groups | One-way ANOVA |  | Figure 5 |  |
|  |  | 127dGA | 48.25 | 4.78 | 6 |  |  |  |  |  |  |  |
|  |  | 143dGA | 54.38 | 8.39 | 5 |  |  |  |  |  |  |  |
|  |  | Newborn | 48.82 | 10.47 | 5 |  |  |  |  |  |  |  |
|  |  | 127dGA TX | 40.81 | 8.73 | 6 | 0.0971 |  | TX vs AMC | t-test |  |  |  |
|  |  | 143dGA T | 44.52 | 7.91 | 5 | 0.0924 |  | TX vs AMC | t-test |  |  |  |

*You may use multiple lines for the same question to indicate multiple comparisons

** Authors may wish to make the text bold where p is considered significant against a stated confidence limit
